# Editorial: Natural compounds/products and livestock productivity: enhancing antioxidant levels, gut health, mitigating greenhouse gas emissions, and disease control, volume II

**DOI:** 10.3389/fvets.2026.1867705

**Published:** 2026-05-21

**Authors:** Moyosore Joseph Adegbeye, Sadarman Sadarman, RaviKanthReddy Poonooru, Edwin Rafael Alvarado-Ramírez, Yeni Widiawati

**Affiliations:** 1 University of Africa, Sagbama, Bayelsa, Nigeria; 2 Department of Animal Science, Faculty of Agriculture and Animal Science, Universitas Islam Negeri Sultan Syarif Kasim Riau, Pekanbaru, Riau, Indonesia; 3 Animal Feed and Nutrition Modelling, Research Group, Faculty of Animal Science, IPB University, Bogor, Indonesia; 4 University of Missouri, Columbia, SC, United States; 5 Faculty of Veterinary Medicine and Zootechnics, Autonomous University of Tamaulipas, Victoria City, Mexico; 6 National Research and Innovation Agency (BRIN), Cibinong, Indonesia

**Keywords:** antioxidant, herbal, immunity, microbiome, natural compounds, plant extract, polyphenol, post-biotics

## Introduction

Plant-derived compounds possess both nutritional and nutraceutical properties that can benefit the livestock production sector, including applications in animal drug manufacturing. This editorial aims to compile and synthesize information demonstrating how natural compounds and phytogenic feed additives can be effectively utilized as ingredients or supplements and how they can be properly integrated into livestock systems. Documenting these applications is essential for conserving valuable species for future use. This Research Topic, developed as Volume II following the success of its previous edition, aims to compile and synthesize recent advances in the application of natural compounds in livestock production systems. A total of 22 manuscripts were received, of which 16 were accepted, including review articles, mini-reviews, and original studies based on *in vitro, in vivo*, and *in silico* approaches. The accepted works cover a wide range of animal species, including ruminants such as goats and cattle, monogastrics such as broilers, ducks, and laying hens, as well as pseudo-ruminants such as rabbits and camels, under both intensive and extensive production systems. About 22 studies were received and only 16 were accepted and they came from United States, Indonesia, Brazil, Tunisia, Peru, and Saudi Arabia (1 each), Mexico (2 submissions), and China (8 submissions) reflecting the global interest in the use of natural compounds as a strategy to improve livestock productivity and sustainability.

The investigation by the various authors include processes such as Green synthesis of compounds, nano/microencapsulation of feed additives; extracts from pomegranate peel, and *Morinda citrifolia*; materials like walnut green husk; seaweed (*Eucheuma cottonii*); *Verbena officinalis* and *Polygonum cuspidatum*; brown mushroom stem waste; *Trollius chinensis Bunge* residue; and bitter almond. Supplementation strategies included yeast selenium, isochlorogenic acid derived from Stevia, and yeast-based post-biotics. Researchers recommended the use of hydroxytyrosol (present in olives), *Caesalpinia coriaria*, and yeast and its culture. Under this approach, the studies address key aspects such as productive performance, antioxidant status, gut health, microbial modulation, methane mitigation, immune function, and metabolic responses in serum and brain, providing a comprehensive view of the role of natural compounds in sustainable livestock production (Table 1).

**Table 1:** Summary of the influence of natural products in livestock.

Natural products/compounds	Species	Effect
Ethanolic extract of *Morinda citrifolia* leaf	Broiler	Decreased *Escherichia coli* and *Staphylococcus aureus*. Improved performance and gut histology
*Verbena officinalis* and *Polygonum cuspidatum*	Ducks	Improved final body weight, increased serum IgG and IgM levels, improved Brain neurochemical profiles while influencing microbiota-gut-brain axis.
Bitter almond	Rongde black-feathered small-sized layer hens	Higher laying rate, improved antioxidant, immune functions, and intestinal morphology. Modified the cecal microbial community.
Isochlorogenic acid (ICGA),	Piglet	Elevated total antioxidant capacity, increased abundance of beneficial *Bacteroidota* and *Prevotella*, while reducing pathogenic taxa, reduced diarrhea incidence, enhanced nutrient digestibility, and strengthened humoral immunity.
*Trollius chinensis* Bunge residue	Rabbit	Improved survival rate, enhanced carcass yield, increased jejunal villus height and villus/crypt ratio in rabbits. Increased the expression of tight junction proteins while reducing pro-inflammatory cytokines.
Yeast-Selenium	Mongolian cashmere goats	Increased levels of glutathione peroxidase, superoxide dismutase, immune capacity observed in blood and seminal plasma. Increased the abundance of bacteria in the rumen.
*S. cerevisiae* post-biotic	Cattle	Prevent gingivitis progression and higher average daily gain
*Agaricus bisporus* stem residues	Poultry	Maintained productive performance in poultry, reducing pollutant gas emissions and improved blood biochemical parameters

## Findings

### In vitro

Several studies highlighted the potential of natural compounds to modulate ruminal fermentation and reduce methane emissions, contributing to both feed efficiency and environmental sustainability. In *in vitro* and *in silico* studies Sheikh et al. noted that due to a changing climate, camels represent a practical livestock option in the Middle East. However, as foregut fermenters (pseudo-ruminants), camels are capable of producing methane, and an increase in their population could elevate methane emissions (1). Based on *in vitro* and *in silico* experiments, it was demonstrated that the dietary inclusion of 0.5 or 1 g/kg of pomegranate peel extract improved nutrient digestibility and ruminal fermentation efficiency, while simultaneously reducing methane production. These effects were mainly attributed to the action of punicalagin, which showed high binding affinity to key enzymes involved in methanogenesis, such as succinate dehydrogenase and F420H_2_ oxidase, suggesting a direct biochemical mechanism of inhibition. Widiawati et al. reported that supplementation with *Eucheuma cottonii* at levels up to 8% on different forage substrates and agricultural by-products modified fermentation dynamics, increased microbial protein synthesis, and reduced methanogen abundance. In addition, substrate type significantly influenced fermentation efficiency, with corn stover showing higher gas production and digestibility efficiency, although with a lower relative proportion of methane in total gas. Microbial analysis showed that rice straw 8% seaweed group exhibited lower methanogen abundance and increased *Rikenellaceae_RC9 gut group* and *Ruminobacter*. These results indicate that both phytochemical compounds and marine resources can act as direct modulators of ruminal fermentation, with implications for mitigating enteric emissions.

### In vivo

#### Monogastric

##### Poultry

Due to their young age, broilers are predisposed to various illnesses, microbial dysbiosis, and opportunistic pathogens, often necessitating antibiotic use to minimize these challenges. Paredes-López et al. showed that an ethanolic extract of *Morinda citrifolia* leaf decreased the abundance of *Escherichia coli* and *Staphylococcus aureus*. A daily dose of 11.0 mg/kg body weight MCEE in the diet improved performance indices by modulating gut microbiota and histology in broiler chickens. In ducks, Zhu et al. reported that treatments with both *Verbena officinalis* and *Polygonum cuspidatum* significantly improved final body weight, increased serum IgG and IgM levels, and decreased feed conversion rates. Correlation analysis indicated associations between changes in gut microbiota (*Villanella, Anaerosporobacter, Anaerofustis, Flavonifractor*) and brain metabolites (GABA, Ach, Glutamate), suggesting a potential influence of these herbs through the microbiota-gut-brain axis. Supplementation with these herbs shows promise as functional feed additives in duck production by enhancing growth performance, immunity, and brain neurochemical profiles. Chen et al. investigated bitter almond, a natural plant-derived additive, in Rongde black-feathered small-sized layer hens. Diets containing 0.5 g/kg bitter almond resulted in a higher laying rate, daily feed intake, and Haugh unit, alongside improved antioxidant (T-AOC, GSH-Px) and immune functions (IgA) compared to the control. The 0.5 g/kg dosage also produced the most pronounced improvement in intestinal morphology. Bitter almond powder modified the cecal microbial community structure, notably resulting in a significant decrease in the abundance of *Negativibacillus* in the 0.75 g/kg group. The abundance of *Negativibacillus* was significantly positively correlated with levels of IgA, IgM, and GSH-Px. A dosage of 0.5 g/kg was determined to be optimal for improving production performance without indicating potential toxicity risks.

##### Hindgut fermenters

###### Pig

Wang et al. investigated isochlorogenic acid (ICGA), a polyphenolic compound found in plants with demonstrated antioxidant, antibacterial, and anti-inflammatory properties (2). In weaned piglets, supplementation with ICGA at 200 mg/kg elevated total antioxidant capacity (T-AOC) by 75.78% and up-regulated IgA and IgM levels. Microbiota analysis indicated that ICGA increased the abundance of beneficial *Bacteroidota* and *Prevotella*, while reducing pathogenic taxa such as *Peptostreptococcaceae, Proteobacteria*, and *Staphylococcus*. Dietary ICGA at 200–400 mg/kg effectively reduced diarrhea incidence, enhanced nutrient digestibility, improved antioxidant capacity, strengthened humoral immunity, and positively modulated gut microbiota.

###### Rabbit

*Trollius chinensis* Bunge is a perennial herb valued for its anti-inflammatory, antioxidant, antibacterial, and antiviral properties, attributed to compounds like flavonoids, organic acids, and alkaloids. Its low toxicity and safety in animal feed have been confirmed (3). Deng et al. showed that graded levels of *Trollius chinensis* Bunge residue (TCBR) at 2.0% (TCBR2) improved survival rate, reduced feed conversion ratio, enhanced carcass yield and antioxidant capacity, and increased jejunal villus height and villus/crypt ratio in rabbits. TCBR2 also significantly increased the expression of tight junction proteins Occludin and ZO-1 while reducing pro-inflammatory cytokines TNF-α and IL-8. 16S rRNA analysis revealed elevated levels of *Bacteroidota, Akkermansia, Clostridium, and Succiniclasticum* in the TCBR2 group, highlighting the potential of low-dose TCBR as a feed additive for rabbits.

##### Ruminants

###### Goat

Yang et al. recommended yeast and its culture for regulating intestinal antioxidant defense in ruminants. Wen et al. reported that supplementing 24-month-old Inner Mongolian cashmere goats with yeast selenium at 0.4 mg/d for 30 days significantly increased levels of glutathione peroxidase (GSH-Px), superoxide dismutase (SOD), IgA, and IgG in blood and semen compared to the control group. Yeast selenium mainly increased the abundance of *Bacteroidales_RF16_group, p-251-o5, UCG-004*, and *UCG-010* in the rumen. Correlation analysis showed that these bacterial groups significantly affected rumen microbiota and fermentation parameters and contributed to the increased antioxidant and immune capacity observed in plasma and seminal plasma. Thus, yeast selenium supplementation can support healthy breeding practices in cashmere goats.

###### Cattle

Controlling oral diseases in ruminants remains a challenge, as traditional oral hygiene practices are unfeasible. This highlights the need for alternative approaches, such as modulating local inflammatory responses through systemic or nutritional routes (4). Gingival inflammation in grazing cattle is often overlooked, and effective control strategies are limited. Saraiva et al. reported that among treatments including a protein-energy supplement with *S. cerevisiae* post-biotic, a standard protein-energy supplement, and a mineral supplement (control), only the post-biotic-supplemented group maintained stable gingivitis frequency throughout the trial. The other groups exhibited a significant increase in gingivitis. Furthermore, post-biotic-supplemented heifers achieved higher average daily gain (ADG) than those receiving the standard or mineral supplements. While post-biotic supplementation did not eliminate gingivitis occurrence, it helped prevent its progression and improved weight performance, representing a promising nutritional strategy for maintaining oral health and productivity in sustainable grazing systems.

##### Feed resources and future strategies in the use of natural compounds

###### Herbals

Cipriano-Salazar et al. highlighted the benefits of *C. coriaria* in livestock production systems, including reduced gastrointestinal parasitism, growth promotion, decreased drug residues in animal products, and mitigation of greenhouse gas emissions. These effects are attributed to its rich content of polyphenolic compounds and other phytochemicals, including tannins, terpenoids, phenols, coumarins, quinones, flavonoids, saponins, carbohydrates, proteins, glycosides, cardiac glycosides, anthraquinones, and steroids. Similarly, Gao et al. reported on the potential of hydroxytyrosol, a polyphenol compound present in olive leaves and fruits, as a natural antioxidant and a promising new feed additive. Advances in biotechnology and animal nutrition are expanding the possibilities for applying natural compounds in livestock systems (5), and the included studies also demonstrate that technological development is key to maximizing their potential. Nefzi et al. recommended that many antioxidants should be green-synthesized to enhance sustainability, stability, and bioavailability in animal feed. Unlike conventional extraction methods, green-synthesized antioxidants derived from plant extracts, essential oils, and agro-industrial by-products offer improved oxidative stability, reduced toxicity, and enhanced bioactivity. Their incorporation into livestock nutrition supports environmentally friendly production practices, aligning with sustainable agriculture and consumer demand for natural animal products. One challenge with additives and supplements is excessive supply to animals to ensure adequate delivery to target organs, leading to excretion of surplus amounts and potential environmental pollution. However, advancements in nutrition, microbiology, and omics sciences offer novel strategies to improve practical efficacy, such as nanotechnology, specifically micro- and nanoencapsulation techniques. Botia Carreño et al., described that these technologies protect bioactive compounds from degradation, provide controlled release, enable targeted delivery to specific absorption sites, and improve probiotic survival and the bioavailability of bioactive compounds.

###### Ingredients

The use of agro-industrial by-products and non-conventional biomass has been proposed as a strategy to improve the sustainability of livestock systems and is consolidating as a relevant approach in sustainable animal nutrition. Abulaiti et al. demonstrated that the inclusion of walnut green husk (30 g/kg) in corn silage improved nutritional quality and digestibility (dry matter, crude protein, and neutral detergent fiber), in addition to promoting lactic acid bacteria and reducing undesirable compounds such as organic acids and ammonia nitrogen. WGH inclusion improved the abundance of *Firmicutes_D* and reduced the abundances of *Bacteroidota* and *Proteobacteria*. The 30 g/kg treatment exhibited the highest relative abundance of dominant bacteria *Firmicutes_D, Lactiplantibacillus, Levilactobacillus*, and *Lacticaseibacillus*. Mushroom species are fungi that can be cultivated on agricultural wastes like sawdust and oil palm bunch. Abdel-Wareth et al. showed that partial replacement (2–6%) of conventional protein sources with *Agaricus bisporus* stem residues maintained productive performance in poultry, while reducing pollutant gas emissions and improving blood biochemical parameters, supporting its value as a functional protein alternative. These shows that the valorization of agro-industrial residues not only contribute to biomass recycling, but also improved nutritional efficiency and mitigates environmental impacts in animal production, as reported in previous studies.

## Conclusion

A promising future lies ahead for the various applications of natural compounds in livestock production, driven by the vast diversity of plants and natural compounds across different continents. The studies included in this Research Topic demonstrate that natural compounds represent a multifunctional strategy to improve livestock production by integrating effects on ruminal fermentation, microbiota modulation, antioxidant status, and productive performance, while simultaneously contributing to optimizing biological efficiency and mitigating environmental impact, particularly through the reduction of methane emissions and the use of non-conventional resources. In this way, the development of technologies such as green synthesis and micro- and nanoencapsulation emerges as a key element to enhance the stability, bioavailability, and efficacy of these compounds, while the valorization of agro-industrial by-products reinforces the role of the circular economy in animal nutrition, constituting a viable pathway toward more sustainable, resilient, and efficient livestock systems.
